# Preferences in the Willingness to Download an mHealth App: Discrete Choice Experimental Study in Spain, Germany, and the Netherlands

**DOI:** 10.2196/48335

**Published:** 2023-12-25

**Authors:** Frans Folkvord, Nadine Bol, Giacomo Stazi, Lutz Peschke, Francisco Lupiáñez-Villanueva

**Affiliations:** 1 Tilburg School of Humanities and Digital Sciences Tilburg University Tilburg Netherlands; 2 PredictBy Barcelona Spain; 3 Open Evidence Barcelona Spain; 4 Department of Communication and Design Bilkent University Ankara Turkey; 5 Department of Information and Communication Science Universidad Oberta de Catalunya Barcelona Spain

**Keywords:** mHealth adoption, discrete choice task, mobile apps, self-monitoring, willingness, mobile health app, mobile app, mobile health, mHealth, adoption, mHealth tools, health care cost, effectiveness, mobile phone

## Abstract

**Background:**

Despite the worldwide growth in mobile health (mHealth) tools and the possible benefits for both patients and health care providers, the adoption of mHealth is low, and only a limited number of studies have examined the intention to download mHealth apps.

**Objective:**

In this study, we investigated individuals’ preferences in the adoption of a health app.

**Methods:**

We conducted a discrete choice experimental study in 3 countries (Spain: n=800, Germany: n=800, and the Netherlands: n=416) with 4 different attributes and levels (ie, price: €1.99 vs €4.99 [a currency exchange rate of €1=US $1.09 is applicable] vs for free, data protection: data protection vs no information, recommendation: patients’ association vs doctors, and manufacturer: medical association vs pharmaceutical company). Participants were randomly assigned. For the analyses, we used the conditional logistic model separately for each country.

**Results:**

The results showed that price and data protection were considered important factors that significantly increased the probability to download an mHealth app. In general, the source of the recommendation and the manufacturer affected the probability to download the mHealth app less. However, in Germany and the Netherlands, we found that if the app was manufactured by a pharmaceutical company, the probability to download the mHealth app decreased.

**Conclusions:**

mHealth tools are highly promising to reduce health care costs and increase the effectiveness of traditional health interventions and therapies. Improving data protection, reducing costs, and creating sound business models are the major driving forces to increase the adoption of mHealth apps in the future. It is thereby essential to create trustworthy standards for mobile apps, whereby prices, legislation concerning data protection, and health professionals can have a leading role to inform the potential consumers.

## Introduction

### Background

One of the main underlying goals of mobile health (mHealth) is to improve the quality of and access to care and reduce the costs related to health care, such as the implementation of mHealth apps in remote health care delivery [1]. mHealth apps are computer programs designed to run on a mobile device (eg, smartphone and tablet) to support health and health-related behavior [2]. Research has shown that mHealth could improve health and well-being worldwide by lowering health care costs, improving the quality of health care, and promoting behavior change to strengthen the prevention of diseases (including chronic diseases). Although these new possibilities promise several benefits for improving health and a healthy lifestyle, the actual adoption and long-term use of mHealth apps is rather low and lagging behind their potential [3]. As a result, despite the promising effects of mHealth apps on health monitoring and improvement [4], there is currently insufficient evidence to effectively inform the implementation and scale-up of mHealth apps. Therefore, it is important to truly understand what individual preferences predict the willingness to download mHealth apps [5].

As mobile technologies are rapidly developing as a method for delivering behavior change interventions, health communication researchers need to improve their understanding of how to advance current theories such that we can leverage and maximize the potential of the ubiquity, adaptability, and affordability of mHealth apps in changing health behaviors [6]. The first step in this endeavor is to understand the impact of various mHealth apps’ attributes in explaining people’s willingness to download mHealth apps. Considering the limited understanding of the general cognitive motivators that trigger people’s adoption of mHealth apps, it is important to examine which attributes of mHealth apps are preferred in the adoption of an mHealth app [7]. Intrinsic motivation to use such an mHealth app is considered a strong predictor of actual adoption and use [6]. Without proper comprehension of the cognitive motivators that explain mHealth apps’ adoption and use, it will be very difficult to establish the effectiveness of mHealth apps and fully understand individuals’ use of such apps.

### Attributes Explaining Preferences for the Adoption of mHealth Apps

The rise of the mHealth apps market threatens to change in way substantial amounts of health data will be managed in the future. Currently, there is a paradigm shift from mainframe systems located in the facilities of health care providers to apps on mobile phones and data stored in shared cloud services [8]. Importantly, attributes such as price and level of privacy of health apps affect the levels of adoption. More specifically, privacy perceptions are related to the use of mHealth apps, meaning that people with more concerns about the secondary use of their personal data were less likely to use certain mHealth apps [9]. Furthermore, free mHealth apps are often appealing to consumers; however, such apps mostly contain other business models, such as advertising or selling of personal data (including health data) [10,11].

Furthermore, health care providers are considered the gatekeepers of health care delivery [12]. Along with the growth of consumption of online platforms, reviews and recommendations have become an important source of information that assists consumers to make purchase decisions [13]. Zhang et al [13] have developed a heuristic-systematic model to examine the influence of online reviews and recommendations on consumption behavior. They showed that the informativeness and persuasiveness of reviews and recommendations are important attributes that enhance the argument quality to decide to consume the product. People decide to consume a certain product based on heuristic information processing, which means that people only consider 1 or a few informational cues and form a judgment based on these cues [14]. In a recent study, COVID-19–tracking apps and health care apps of different countries were analyzed with the help of a qualitative content analysis according to their reward mechanisms based on the Mobile Application Rating Scale approach for assessment [15]. The analysis included a correlation of different rewards for voluntary participation. The Mobile Application Rating Scale approach consists of engagement, functionality aesthetics, and information quality. It could be understood that motivational strategies in terms of gamification and tools for individual knowledge exchange reduce the inhibition threshold of downloading and using health care apps and COVID-19–tracking apps.

Based on the least effort principle, people prefer to use less cognitive effort in general and to spend much effort only when they have to [16]. For example, if an app is recommended by an expert, for instance by a doctor, people will perceive this advice as credible in their decision to adopt and use it [17]. There are at least 2 reasons why a doctor’s recommendation for an mHealth app can be considered a strong enforcer for patients to adopt and use digital health technologies. First, doctors are considered experts in their field of work and therefore have more influence than nonexperts, in particular, because they also know the patients and their interests quite well [18-20]. Second, doctors’ professionalism forces them to act upon patients’ interests first; most patients, therefore, trust the doctor more than other actors [21].

Finally, the manufacturer of an mHealth app can be considered a heuristic in consumers’ adoption of an mHealth app. There is a reason to assume that this may be the case, as the pharmaceutical industry has struggled with its public image over the past few decades. Pharmaceutical companies need to negotiate a tension between, on the one hand, striving for optimal health care and, on the other hand, striving for profit [22]. In the eyes of the public, it is not always clear that the pharmaceutical industry has patients’ interests at heart [23]. Therefore, if an mHealth app is manufactured by a medical association, people probably will prefer this more than when the app is manufactured by the pharmaceutical industry.

### This Study

Therefore, we conducted a large-scale discrete choice experiment (DCE) in 3 different countries (Spain, Germany, and the Netherlands), whereby we manipulated the price (for free vs €4.99 vs €1.99 [a currency exchange rate of €1=US $1.09 is applicable]), data protection (data protection vs no information), recommendation (recommended by patients’ association vs doctors), and manufacturer (medical association vs pharmaceutical company) and assessed the likelihood to adopt the mHealth app. This study explores differences between 3 European countries with varying cultures and health care infrastructures: Spain, Germany, and the Netherlands. In short, Spain has a national health system that is an agglomeration of public health services established by the general health law. The vast majority of final providers of care are part of the regional health service structure and are not autonomous legal entities. In Germany, there is a statutory health insurance system, with 131 competing statutory health insurers (“sickness funds” in a national exchange), where high-income citizens can opt out of private coverage. In the Netherlands, there is a statutory health insurance system, with universally mandated private insurance (national exchange), where the government regulates and subsidizes insurance for people who need it. Considering the differences in health care systems, the experiment was conducted in these 3 countries.

We aim to assess a comprehensive assessment of the preferences of people for the adoption of an mHealth app. To our knowledge, no previous study has attempted to disentangle preferences for the adoption of an mHealth app using a DCE. Insights into attributes that could influence whether individuals are more likely to adopt an mHealth app should therefore be very helpful in informing product development and pathway redesign for future mHealth technologies.

## Methods

### Design

In this study, we used a DCE in an online questionnaire. In comparison with other preference elicitation methods, a DCE can quantify the relative importance of different attributes that characterize a new or existing product or service, identifying which attributes people prioritize or accept and which they may be willing to exchange to maximize their use [24]. A DCE requires participants to choose between competing scenarios, for example, costs, described in terms of a particular attribute (eg, price) and a range of levels (eg, for free, €1.99, and €4.99) and to compare these against an alternative scenario. By providing participants with different attributes and levels within these attributes and subsequently asking the participant to make a decision on which option they prefer over the other, researchers are able to detect the most preferred combination of options in the decision-making of a participant. DCE studies are very relevant because they allow a direct cognitive assessment of relative preferences for various existing and hypothetical new service configurations or treatment approaches. Furthermore, by using a DCE, it is possible to directly assess the complexity of human decision-making by providing the participant with small changes in the options that they are exposed to.

The online questionnaire that we used in this study adopted a main effects design using all attributes included in the study. In the main effects design, all possible levels are included, and each pair of levels occurs equally often. A full factorial design was ruled out in favor of a fractional factorial design because a full factorial design would have contained too many possible alternatives that would have been unmanageable in practice for individuals to complete or for a blocked questionnaire format to handle.

The use of an online questionnaire provided completion time data to support the internal validity checks and enabled an accurate record of the time taken to complete the surveys. Two rounds of cognitive testing (n=48) were undertaken in the Netherlands to check participants’ comprehension of information when making choices. These pretests confirmed that a study based on the questionnaire was acceptable and understandable for participants after some minor revisions in the explanation of the task. The 4 attributes (price, data protection, recommendation, and manufacturer) and levels selected for inclusion in the DCE are shown in Textbox 1.

**The attributes and levels selected for the discrete choice experiment.**

**Price**

- C1: €1.99 (a currency exchange rate of €1=US $1.09 is applicable)
- C2: €4.99
- C3: For free

**Data protection**

- D1: Data protection
- D2: No information

**Recommendation**

- P1: This app is recommended by patients’ association
- P2: This app is recommended by doctors

**Manufacturer**

- M1: Medical association
- M2: Pharmaceutical company

### Procedure

All survey participants were informed about the overall study goals and procedures. Only those who agreed to participate in the study gained access to the online survey.

First, participants were asked to provide sociodemographic information, including their age, gender, education, and employment status. Subsequently, an introduction to the DCE questionnaire explained the attributes and levels. A generic pairwise choice with an opt-out question (“I would not download the app”) was selected for the questionnaire design. Participants were presented with a series of choice sets for which there were 3 responses: “Option A,” “Option B,” and “I would not download the app.” A sample choice set is illustrated in Textbox 2.

**A “mock-up” of the discrete choice task.**

**Option A**

- “The price for the app is €4.99” (a currency exchange rate of €1=US $1.09 is applicable)
- “There is no information given about what is done with the data that you enter and is saved by the app”
- “The app is recommended by Patient’s association”
- “The app is manufactured by a pharmaceutical company”

**Option B**

- “The app is for free”
- “There is no information given about what is done with the data that you enter and is saved by the app”
- “The app is recommended by Doctors”
- “The app is manufactured by a pharmaceutical company”

**Not download**

- “I would not download the app”

### Participants

For this study, we used data collected from 3 different countries (Spain, Germany, and the Netherlands). This study was part of a larger project, whereby multiple experiments were conducted. After participants finished the discrete choice experiment, they also participated in a separate experiment that is reported in a separate paper. The data in Spain (n=800) and Germany (n=800) were collected through an online survey administered by a Spanish professional research company, well known for managing large sampling pools in Europe. The sample was chosen through a proportionate stratified sampling method, considering gender and age. The data in the Netherlands (n=416) were collected through snowball sampling by sharing a link to the questionnaire via social media platforms.

### Ethical Considerations

This study was approved by the ethical committee of the Universitat Oberta de Catalunya (number TCApp.2017-01). Written and informed consent was obtained from all participants. All study data are anonymous. Participants from Spain and Germany received a small financial compensation (€10 per person) for participating in the study. Participants in the Netherlands did not receive financial compensation for participating in the study. Through signing the informed consent, participants were ensured that their data would remain confidential, and they were told that they could cease participation at any moment.

### Statistical Analyses

We analyzed the choice experiment data using a conditional logit model estimated by means of the *Clogit* R software (R Foundation for Statistical Computing). The data structure required for the estimation contains 1 row for each alternative that the decision maker had to consider in each choice scenario. For each alternative, the dependent variable is coded as a dummy variable equal to 1 if the respondent chose that option. Since each participant faced 18 choice scenarios with 3 alternatives (2 apps and the opt-out option), the data set contains 43,200 rows for Spain and Germany (800×18×3) and 22,464 rows for the Netherlands (416×18×3).

We specify the deterministic component of the utility function as follows:

*V_ij_* = *β*_1_ alternative specific constant + *β*_2_ price_1.99 + *β*_3_ price_4.99 + β_4_ data protection + β_4_ doctor recommendation + β_5_ pharmaceutical_i. Individual(1)

where each regressor represents an indicator variable for whether that specific feature was present in the app. The alternative specific constant represents which of the options in each choice task is the opt-out option. Thus, the coefficient associated with it estimates whether individuals were more likely to choose not to download the app. The option “not to download” the app was included because we want to assess the preference of the participants in selecting to download the app while not forcing them to choose that.

## Results

The participant characteristics are shown in Table 1. The descriptive information shows that the participants from the Netherlands are mostly a younger (male) student population with higher levels of education; lower financial status; lower scores on health consciousness, health information orientation, and visits to the doctor; and a better health status than the participants from Spain and Germany. Furthermore, in Germany and Spain, the gender of participants is equally distributed. The same applies to average age, educational level, and employment status.

Table 2 shows the results of the conditional logistic model separately for each country. Results are presented in terms of odds ratios (OR). Thus, values larger than 1 represent an increase in the likelihood of downloading the app compared to the reference category.

First, respondents were more likely to download the apps when they were for free. Nonetheless, compared to Spain and Germany, participants in the Netherlands were less sensitive to a priced app when the cost was €1.99 (OR 0.943, 95% CI 0.866-1.026 in the Netherlands; OR 0.249, 95% CI 0.234-0.265 in Spain; and OR 0.255, 95% CI 0.239-0.271 in Germany). Next, in all countries, participants valued positively the apps that ensured data protection (OR 2.679, 95% CI 2.530-2.836 in Spain; OR 1.473, 95% CI 1.391-1.559 in Germany, and OR 2.045, 95% CI 1.899-2.203 in the Netherlands). With regards to the type of endorsement received by the app, we found that Spanish and German respondents preferred apps recommended by patients’ associations (OR 0.750, 95% CI 0.706-0.795 in Spain and OR 0.748, 95% CI 0.705-0.794 in Germany), while Dutch respondents preferred apps recommended by doctors (OR 1.100, 95% CI 1.018-1.188). The attribute representing the manufacturer also had a different impact across countries. While Spanish respondents slightly preferred apps developed by pharmaceutical companies (OR 1.028, 95% CI 0.971-1.087), German and Dutch respondents were more likely to download apps produced by medical associations (OR 0.608, 95% CI 0.574-0.644 in Germany and OR 0.513, 95% CI 0.476-0.553 in the Netherlands). Last, we found that Dutch respondents were more likely to choose to not download the app compared to Spanish and German participants (OR 1.516, 95% CI 1.366-1.682 in the Netherlands; OR 0.675, 95% CI 0.627-0.728 in Spain; and OR 0.701, 95% CI 0.651-0.755 in Germany).

**Table 1:** Descriptive information about the participants per country.

Characteristic		Spain (n=800)	Germany (n=800)	The Netherlands (n=416)
Gender: women, n (%)		400 (50)	400 (50)	159 (38.2)
Age (years), mean (SD)^a^		41.60 (13.32)	45.92 (15.09)	28.92 (14.86)
**Educational level, n (%)^a^**				
	Primary education	26 (3.3)	224 (28)	8 (1.9)
	High school diploma	200 (25)	285 (35.6)	57 (13.7)
	Some years of university	136 (17)	59 (7.4)	105 (25.2)
	University degree	335 (41.9)	170 (21.3)	175 (42.1)
	Postgraduate degree	103 (12.9)	62 (7.8)	71 (17.1)
**Employment status, n (%)^a^**				
	Employed or self-employed	586 (73.3)	468 (58.5)	161 (38.7)
	Unemployed	64 (8)	36 (4.5)	9 (2.2)
	Student	55 (6.9)	55 (6.9)	238 (57.2)
	Retired	60 (7.5)	178 (22.3)	4 (1)
	Not working due to illness or disability	12 (1.5)	27 (3.4)	2 (0.5)
	Another nor in the labor force	23 (2.9)	36 (4.5)	2 (0.5)
**Difficulty paying bills, n (%)^a^**				
	Most of the time	123 (15.4)	62 (7.8)	21 (5)
	From time to time	293 (36.6)	199 (24.9)	78 (18.8)
	Almost never ever	375 (46.9)	525 (65.6)	291 (70)
	No answer	9 (1.1)	14 (1.8)	26 (6.3)
**Health app use, n (%)**				
	No use	420 (52.5)	565 (70.6)	285 (68.5)
	1 time	105 (13.1)	71 (8.9)	37 (8.9)
	2 times	82 (10.3)	73 (9.1)	28 (6.7)
	3 times	71 (8.9)	36 (4.5)	25 (6)
	4 times	33 (4.1)	10 (1.3)	11 (2.6)
	5 times	22 (2.8)	13 (1.6)	4 (1)
	6 or more times	67 (8.4)	32 (4)	26 (6.3)
Health consciousness, mean (SD)^a^		4.06 (0.78)	3.82 (0.85)	3.43 (0.87)
Health information orientation, mean (SD)^a^		3.63 (0.83)	3.30 (0.96)	2.79 (1.01)
eHealth literacy, mean (SD)^a^		3.51 (0.93)	3.50 (0.94)	3.08 (1.03)
**Visit doctor last year, n (%)^a^**				
	Never	78 (9.8)	95 (11.9)	102 (24.4)
	Once	156 (19.5)	114 (14.2)	87 (20.9)
	Twice	170 (21.3)	151 (18.9)	80 (19.2)
	3 times	128 (16)	104 (13)	54 (13)
	4 times	86 (10.8)	97 (12.1)	31 (7.5)
	5 times or more	182 (22.8)	236 (29.5)	57 (13.7)
**Health in general, n (%)^a^**				
	Very bad	1 (0.1)	11 (1.4)	3 (0.7)
	Bad	21 (2.6)	78 (9.8)	6 (1.4)
	Neither good nor bad	129 (16.1)	204 (25.5)	41 (9.9)
	Good	461 (57.6)	391 (48.9)	223 (53.6)
	Very good	188 (23.5)	116 (14.5)	143 (34.4)

^a^*P*<.001.

**Table 2:** Conditional logistic model for the discrete choice experiment outcomes among participants in Spain, Germany, and the Netherlands.

Variable		Dependent variable: choice		
		Spain	Germany	The Netherlands
**Price €1.99^a^**				
	OR^b^ (95% CI)	0.249^c^ (0.234-0.265)	0.255^c^ (0.239-0.271)	0.943^c^ (0.866-1.026)
	SE	0.032	0.032	0.043
**Price** **€** **4.99**				
	OR (95% CI)	0.125^c^ (0.116-0.135)	0.139^c^ (0.128-0.150)	0.586^c^ (0.533-0.645)
	SE	0.039	0.039	0.049
**Data protection**				
	OR (95% CI)	2.679^c^ (2.530-2.836)	1.473^c^ (1.391-1.559)	2.045^c^ (1.899-2.203)
	SE	0.029	0.029	0.038
**Doctor recommendation**				
	OR (95% CI)	0.750^c^ (0.706-0.795)	0.748^c^ (0.705-0.794)	1.100^c^ (1.018-1.188)
	SE	0.030	0.030	0.039
**Pharmaceutical manufacturer**				
	OR (95% CI)	1.028^c^ (0.971-1.087)	0.608^c^ (0.574-0.644)	0.513^c^ (0.476-0.553)
	SE	0.029	0.029	0.038
**Alternative specific constant**				
	OR (95% CI)	0.675^c^ (0.627-0.728)	0.701^c^ (0.651-0.755)	1.516^c^ (1.366-1.682)
	SE	0.038	0.038	0.053
Observations		43,200	43,200	22,458
*R* ^2^		0.113	0.117	0.047
Maximum possible *R*^2^		0.697	0.697	0.697
Log likelihood		–23,165.480	–23,074.500	–12,852.110
Wald test		4127.660^c^	4488.590^c^	976.660^c^
Likelihood ratio test		5197.001^c^	5378.955^c^	1082.782^c^
Score (logrank) test		4847.258^c^	5021.592^c^	1023.124^c^

^a^A currency exchange rate of €1=US $1.09 is applicable.

^b^OR: odds ratio.

^c^*P*<.01.

## Discussion

### Principal Findings

This study conducted a large-scale DCE in 3 different countries (Spain, Germany, and the Netherlands), whereby we manipulated the levels of price, data protection, recommendation, and manufacturer and assessed preferences for downloading an mHealth app. We aimed to conduct a comprehensive assessment of the preferences of people for the adoption of an mHealth app. A DCE requires participants to choose between competing scenarios and to compare these against an alternative scenario, resulting in a clearer view of people’s preferences.

The results showed that price and data protection were important factors relating to the willingness to download the mHealth app. If the app was for free, participants in Spain, Germany, and the Netherlands were much more likely to download the app compared to when the app had a price of €1.99 or €4.99. Although these prices are fairly low, these factors did not affect the probability to download the health app or not significantly. In addition, in all countries, if the data were protected by European Union legislation, participants were more likely to download the app than when no information was provided.

If the app was recommended by doctors, we found a small negative effect on the probability to download the health app in Spain and Germany and a small positive effect on the probability to download the health app in the Netherlands. In Germany and the Netherlands, we found a small negative effect on the probability to download the health app if it was manufactured by the pharmaceutical association, while in Spain it made no difference.

In general, based on heuristic information processing, people applied the least effort principle to consume a certain product, which means that people consider only 1 or a few informational cues and form a judgment based on these cues [14,16]. This study showed that, in particular, price and data protection were important heuristics for people to decide whether or not they are willing to download an app. Recommendations by experts or the manufacturing of the app were less important, although most people were much less likely to download an mHealth app when it was developed by a pharmaceutical company.

Following the health belief model [25] and the technology acceptance model [26], people will not take health or preventive measures unless they are determined enough to start using mHealth apps because they think it is useful and relevant for them, whereby factors such as price and privacy protection play an important role as barriers or drivers in the adoption of an mHealth app [27]. Therefore, validated guidelines and reliable effectiveness are necessary. The currently available reviews of mHealth apps have predominantly focused on personal impressions, rather than evidence-based clinical trials conducted by researchers and unbiased assessments of clinical performance and data [28]. It would therefore be helpful if health care professionals and institutes and organizations would come up with certain guidelines to help developers to build high-quality and useful apps. These guidelines might include a broad range of categories, such as safety, accuracy, effectiveness, and security. As the popularity of mHealth apps increases, clinicians might therefore wish to prescribe certain apps and access the clinical insights these apps generate.

Improving patient safety (data protection), reducing costs, and creating sound business models are the major driving forces in the adoption of mHealth in the future. It is essential to create standards for mobile apps. Governments, large funders, and industry associations should create and adhere to such standards so that consumers can adapt and use mHealth apps, being confident that such apps are of high quality, take care of data protection, and that prices reflect the value such apps provide.

### Strengths and Limitations

One of the strengths of this study is that we collected data among a large group of participants in 3 different countries, examining preferences for the adoption of an mHealth app. Considering the 3 countries in this study (Spain, Germany, and the Netherlands) have comparable health contexts to other countries within the European Union, we believe the outcomes can also be translated to other countries within and beyond the European Union. To our knowledge, no previous study has attempted to disentangle the strength of preferences for attributes for the adoption of an mHealth app. Insights into attributes that could influence whether individuals are more likely to adopt an mHealth app should therefore be very helpful in informing product development and pathway redesign for future mHealth technologies. Second, we analyzed a great variety of factors within the study to test several attributes that are considered important in predicting and explaining the adoption of mHealth apps. Third, we assessed a great variety of factors to establish cognitive motivators for the adoption of an mHealth app.

The study also had some limitations. First, as the study was conducted online, the internal validity of the exact experiment cannot be guaranteed since it is difficult to tell how serious participants have involved in the experiment. Nonetheless, because the experiment focused on factors that predict the adoption of an mHealth app—which can be considered an online behavior—using an online questionnaire to assess different factors can be considered a valid and reliable method. Second, in Spain and Germany, data were collected by a professional company, paying participants for their participation, while in the Netherlands, the snowballing sample was used without paying the participants. Overall, the results are quite similar between the countries, although we also notice some minor differences in some results that could be due to the different sampling methods.

### Conclusions

Over the last 2 decades, the number of people in the world who use their smartphone for health-related purposes has increased rapidly [29,30]. However, research into the cognitive motivators explaining the adoption of mHealth is still scarce, while adopting an mHealth app is necessary to be effective [31,32]. The insights gathered in this study can be used by diverse stakeholders involved in developing mHealth tools. Improving patient safety (data protection), reducing costs, and creating sound business models are factors that will drive the adoption of mHealth in the future [33]. It is thereby essential to create trustworthy standards and guidelines for mobile apps, whereby prices, legislation concerning data protection, and doctors and patients’ associations can have a leading role in informing potential consumers. Governments, large funders, and industry associations should create and adhere to standards so that mHealth apps can be adopted and used with confidence of the quality and privacy of the data and with prices that are accustomed to what is provided.

## Abbreviations

DCE: discrete choice experiment
mHealth: mobile health
OR: odds ratio

## Data Availability

The data sets generated during and analyzed during this study are available from the corresponding author on reasonable request.

## References

[1] Silva BMC, Rodrigues JJPC, de la Torre Díez I, López-Coronado M, Saleem K (2015). Mobile-health: a review of current state in 2015. J Biomed Inform.

[2] Smit ES, Bol N (2019). From self-reliers to expert-dependents: identifying classes based on health-related need for autonomy and need for external control among mobile users. Media Psychol.

[3] Tomlinson M, Rotheram-Borus MJ, Swartz L, Tsai AC (2013). Scaling up mHealth: where is the evidence?. PLoS Med.

[4] Kumar S, Nilsen WJ, Abernethy A, Atienza A, Patrick K, Pavel M, Riley WT, Shar A, Spring B, Spruijt-Metz D, Hedeker D, Honavar V, Kravitz R, Lefebvre RC, Mohr DC, Murphy SA, Quinn C, Shusterman V, Swendeman D (2013). Mobile health technology evaluation: the mHealth evidence workshop. Am J Prev Med.

[5] Anastasiadou D, Folkvord F, Lupiañez-Villanueva F (2018). A systematic review of mHealth interventions for the support of eating disorders. Eur Eat Disord Rev.

[6] Ng JYY, Ntoumanis N, Thøgersen-Ntoumani C, Deci EL, Ryan RM, Duda JL, Williams GC (2012). Self-determination theory applied to health contexts: a meta-analysis. Perspect Psychol Sci.

[7] Folkvord F, Peschke L, Ağca YG, van Houten K, Stazi G, Roca-Umbert A, Peschke SG, Seyfafjehi S, Gallego A, Gaeta E, Fico G, Karinsalo A, Villanueva FL (2022). Preferences in the intention to download a COVID tracing app: a discrete choice experiment study in the Netherlands and Turkey. Front Commun.

[8] He D, Naveed M, Gunter CA, Nahrstedt K (2014). Security concerns in android mHealth apps. AMIA Annu Symp Proc.

[9] Bol N, Helberger N, Weert JCM (2018). Differences in mobile health app use: a source of new digital inequalities?. Inf Soc.

[10] Sax M, Helberger N, Bol N (2018). Health as a means towards profitable ends: mHealth apps, user autonomy, and unfair commercial practices. J Consum Policy.

[11] Xia R, Rost M, Holmquist LE (2010). Business models in the mobile ecosystem.

[12] Bernstein ML, McCreless T, Côté MJ (2007). Five constants of information technology adoption in healthcare. Hosp Top.

[13] Zhang KZ, Zhao SJ, Cheung CM, Lee MK (2014). Examining the influence of online reviews on consumers' decision-making: a heuristic–systematic model. Decis Support Syst.

[14] Tam KY, Ho SY (2005). Web personalization as a persuasion strategy: an elaboration likelihood model perspective. Inf Syst Res.

[15] Peschke L, Peschke SG, Agca YG, Seyfafjehi S, Dündar I, Aydogdu Y (2022). Analysis of reward mechanism in COVID-19 tracking apps and its impact on voluntary participation of the public in sustainable innovation processes. Turk Rev Commun Stud.

[16] Bohner G, Moskowitz GB, Chaiken S (1995). The interplay of heuristic and systematic processing of social information. Eur Rev Soc Psychol.

[17] Chen S, Chaiken S, Chaiken S, Trope Y (1999). The heuristic-systematic model in its broader context. Dual-Process Theories in Social Psychology.

[18] Gerber BS, Eiser AR (2001). The patient physician relationship in the internet age: future prospects and the research agenda. J Med Internet Res.

[19] Leigh S, Ashall-Payne L (2019). The role of health-care providers in mHealth adoption. Lancet Digit Health.

[20] Peng W, Yuan S, Holtz BE (2016). Exploring the challenges and opportunities of health mobile apps for individuals with type 2 diabetes living in rural communities. Telemed J E Health.

[21] Calnan MW, Sanford E (2004). Public trust in health care: the system or the doctor?. BMJ Qual Saf.

[22] Bauchner H, Fontanarosa PB (2013). Restoring confidence in the pharmaceutical industry. JAMA.

[23] Olsen AK, Whalen MD (2009). Public perceptions of the pharmaceutical industry and drug safety: implications for the pharmacovigilance professional and the culture of safety. Drug Saf.

[24] Lancsar E, Louviere J (2008). Conducting discrete choice experiments to inform healthcare decision making: a user's guide. Pharmacoeconomics.

[25] Ahadzadeh AS, Sharif SP, Ong FS, Khong KW (2015). Integrating health belief model and technology acceptance model: an investigation of health-related internet use. J Med Internet Res.

[26] Yuen KF, Cai L, Qi G, Wang X (2020). Factors influencing autonomous vehicle adoption: an application of the technology acceptance model and innovation diffusion theory. Technol Anal Strateg Manag.

[27] Champion VL, Skinner CS, Glanz K, Rimer BK, Viswanath K (2008). The health belief model. Health Behavior and Health Education: Theory, Research, and Practice.

[28] Powell AC, Landman AB, Bates DW (2014). In search of a few good apps. JAMA.

[29] Gagnon MP, Ngangue P, Payne-Gagnon J, Desmartis M (2016). m-Health adoption by healthcare professionals: a systematic review. J Am Med Inform Assoc.

[30] Putzer GJ, Park Y (2010). The effects of innovation factors on smartphone adoption among nurses in community hospitals. Perspect Health Inf Manag.

[31] Böhm B, Karwiese SD, Böhm H, Oberhoffer R (2019). Effects of mobile health including wearable activity trackers to increase physical activity outcomes among healthy children and adolescents: systematic review. JMIR Mhealth Uhealth.

[32] Marcolino MS, Oliveira JAQ, D'Agostino M, Ribeiro AL, Alkmim MBM, Novillo-Ortiz D (2018). The impact of mHealth interventions: systematic review of systematic reviews. JMIR Mhealth Uhealth.

[33] Chan A, Kow R, Cheng JK (2017). Adolescents’ perceptions on smartphone applications (apps) for health management. J Mobile Technol Med.

